# Bioactive Fillers in Bulk-Fill Composite Resins: A Comprehensive Review of the Effects on Polymerization Shrinkage Behavior and Mechanical Performance

**DOI:** 10.3390/ma19112181

**Published:** 2026-05-22

**Authors:** Vlad Constantin, Ionut Luchian, Ionut Taraboanta, Teona Anamaria Tudorici, Nicoleta Tofan, Florinel Cosmin Bida, Florin Razvan Curca, Dana Gabriela Budala, Dragos Ioan Virvescu, Andrei Georgescu

**Affiliations:** Grigore T. Popa University of Medicine and Pharmacy, 700115 Iasi, Romania

**Keywords:** bioactive fillers, bulk-fill composite resins, polymerization shrinkage, stress development, matrix–filler interaction, ion-releasing systems

## Abstract

Polymerization shrinkage remains a primary cause of marginal failure in posterior composite restorations, contributing to interfacial gap formation and secondary caries development. Bioactive filler technologies represent a paradigm shift, offering simultaneous stress reduction and therapeutic ion release through engineered matrix–filler interactions. This narrative review synthesizes current evidence on how bioactive glass (including 45S5), amorphous calcium phosphate, and surface pre-reacted glass-ionomer fillers modulate polymerization shrinkage dynamics and mechanical performance in bulk-fill systems. These systems exhibit distinct mechanisms of bioactivity, with bioactive glass (45S5) promoting ion release and apatite formation, amorphous calcium phosphate (ACP) enabling rapid calcium phosphate ion delivery for remineralization, and surface pre-reacted glass-ionomer (S-PRG) fillers providing sustained multi-ion release with buffering and antibacterial potential. A comprehensive literature search was conducted in PubMed/MEDLINE, Scopus, and Web of Science for studies published up to June 2025, including experimental investigations and reviews assessing bioactive filler integration, with studies selected based on predefined inclusion and exclusion criteria focusing on relevance and reported outcomes. The available evidence indicates that optimized bioactive formulations reduce shrinkage stress by approximately 25–40%, decreasing from 35–40 MPa in conventional systems to 22–32 MPa in bioactive bulk-fill composites while maintaining flexural strength above 100 MPa and elastic modulus within clinically acceptable ranges (11–13 GPa). However, substantial heterogeneity in filler chemistry, loading protocols, and testing methodologies limits cross-study comparisons. This variability also reflects differences in testing conditions, material compositions, and evaluation protocols across studies.

## 1. Introduction

Resin-based composite materials represent the cornerstone of contemporary restorative dentistry, valued for their superior esthetic properties, reliable adhesive capabilities, and compatibility with minimally invasive treatment paradigms [1,2]. Innovations in filler chemistry, resin matrix formulation, and photopolymerization technology have progressively enhanced clinical performance, extending composite applications from anterior regions into high-stress posterior environments [3,4]. This transition reflects the broader adoption of biomimetic restorative principles, which prioritize preservation of sound tooth structure while maintaining adequate mechanical durability and long-term functional stability [5,6].

Nevertheless, polymerization shrinkage persists as a fundamental material limitation [7]. During photopolymerization, monomer-to-polymer conversion generates volumetric contraction (1.0–3.0% in conventional systems) that produces internal stresses at the tooth–restoration interface, potentially compromising marginal adaptation, initiating interfacial gap formation (50–150 μm in conventional systems), and promoting postoperative sensitivity and microleakage [8,9]. These effects are particularly critical in high C-factor posterior restorations, where stress amplification increases the risk of marginal failure and secondary caries [10,11]. Consequently, shrinkage mitigation remains a primary objective in restorative material development.

Bulk-fill composite resins were introduced to address this challenge through a coordinated technological approach [12]. These materials employ three integrated strategies:

(1) Incorporation of stress-relieving high-molecular-weight dimethacrylates and addition–fragmentation chain-transfer mechanisms [13,14];

(2) Optimized filler loading (70–85% *w*/*w*) and refined particle size distributions that enhance stress distribution and reduce the relative volume of polymerizable matrix [15];

(3) Enhanced material translucency enabling adequate light penetration at depths of 4–5 mm, thereby ensuring uniform polymerization kinetics [16]. Consequently, bulk-fill systems permit single-increment placement while achieving polymerization behavior and mechanical properties comparable to or exceeding conventional incremental techniques, particularly in posterior load-bearing restorations [17,18]. Clinical evidence confirms acceptable marginal adaptation and reduced secondary caries incidence with bulk-fill formulations [19,20].

Concurrently, bioactive fillers have emerged as a complementary technological approach to simultaneously enhance material performance and optimize biological interactions at the tooth–restoration interface [21,22]. Unlike conventional inert filler particles, bioactive fillers release therapeutic ions—including calcium (Ca^2+^), phosphate (PO_4_^3−^), fluoride (F^−^), and strontium (Sr^2+^)—that promote remineralization and local pH buffering, increasing pH from acidic conditions (5.5–6.0) toward physiological levels (7.0–7.5) to inhibit cariogenic processes [23,24]. Furthermore, several bioactive filler systems demonstrate the capacity to modulate bacterial biofilm formation, thereby enhancing interfacial stability in cariogenic environments [25,26]. Contemporary bioactive technologies, including bioactive glass (BAG), calcium phosphate (CaP) systems, and surface pre-reacted glass-ionomer (S-PRG) particles, represent important progress toward functionally interactive restorative materials with integrated therapeutic potential [27,28].

Despite extensive investigation of bulk-fill polymerization behavior and bioactive filler bioactivity, several critical gaps remain: (1) limited comparative data on how bioactive filler incorporation specifically influences polymerization shrinkage kinetics and stress development in bulk-fill systems: specifically whether bioactive fillers reduce shrinkage stress toward 22–32 MPa (as preliminary evidence suggests or whether ionic release merely redistributes stress rather than mitigating net volumetric contraction) [29]; (2) unclear effects on mechanical properties: namely whether bioactive fillers compromise the structural advantages inherent to bulk-fill design, including flexural strength (≥80 MPa), elastic modulus (≥10 GPa), and fracture toughness (≥0.9 MPa·m^1^/^2^) [30], particularly regarding wear resistance in high-stress posterior contacts; (3) insufficient understanding of bioactivity persistence: in particular whether ion-release capacity and biological activity are maintained when bioactive fillers are dispersed within bulk-fill matrices with modified polymerization profiles (extended viscous flow phase or altered gel-point dynamics) and hydrophobic resin chemistry [31]; and (4) lack of standardized methodologies for shrinkage stress evaluation across experimental bioactive bulk-fill systems.

These values reflect differences between conventional and bioactive composite systems and should not be interpreted as inconsistencies, as they depend on variations in material composition, filler type, and testing conditions.

Reported degrees of conversion values for bioactive bulk-fill composites typically range between 55% and 75%, depending on formulation and curing conditions, which may influence both mechanical performance and shrinkage behavior.

Bioactivity in these materials is primarily associated with ion release and remineralization potential; however, this is often accompanied by structural trade-offs, including reduced filler–matrix bonding efficiency, lower cross-link density, and increased water sorption. These factors may lead to decreases in flexural strength, elastic modulus, and long-term mechanical stability, depending on filler type and loading strategy.

Available evidence indicates that bioactive bulk-fill composites consistently exhibit lower polymerization shrinkage stress compared to conventional materials, with values decreasing from approximately 35–40 MPa in conventional systems to 22–32 MPa in bioactive formulations. This comparison should be interpreted as a direct contrast between conventional and modified systems rather than as inconsistencies in reported data. The variability observed across studies is primarily attributed to differences in material composition, filler chemistry, loading strategies, and testing conditions, which significantly influence the magnitude of shrinkage stress values.

**Figure 1:**
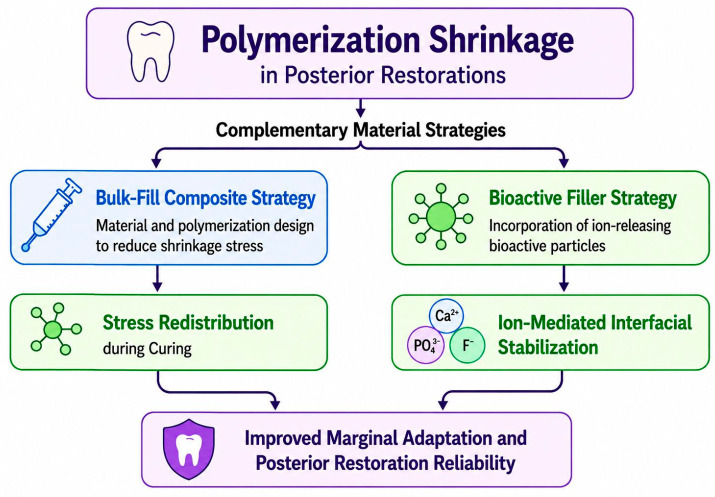
Conceptual framework illustrating complementary material strategies used to mitigate polymerization shrinkage effects in posterior bulk-fill composite restorations through stress redistribution mechanisms and ion-release-mediated interfacial stabilization.

These gaps remain clinically relevant: clarifying the interaction between bioactive fillers and shrinkage behavior could inform development of next-generation posterior materials that simultaneously address polymerization shrinkage (currently 35–40 MPa in conventional systems) while providing therapeutic benefits equivalent to those documented for bioactive systems in anterior applications, as illustrated in Figure 1 below:

Accordingly, this review aims to:

(i) Comprehensively analyze the influence of bioactive fillers on polymerization shrinkage kinetics, stress development, and polymerization behavior in bulk-fill composite resins;
(ii) Synthesize current evidence regarding mechanical performance and clinical implications of bioactive bulk-fill systems for posterior restorative treatment;
(iii) Identify remaining research gaps and propose future investigation directions to optimize bioactive filler integration into high-performance bulk-fill systems.

## 2. Literature Overview and Selection Approach

Synthesizing evidence on bioactive filler systems within bulk-fill composites presents inherent challenges due to heterogeneous experimental designs, variable filler formulations, and disparate testing protocols across published investigations. Bioactive glass, amorphous calcium phosphate, and surface pre-reacted glass-ionomer fillers have been evaluated under inconsistent conditions, complicating direct comparisons.

Quantitative comparisons across the reviewed studies suggest that polymerization shrinkage values generally range between approximately 1.5% and 3.5% for bulk-fill composites, while shrinkage stress typically falls within the range of 20–35 MPa, depending on the formulation and testing methodology. Flexural strength values are commonly reported above 100 MPa, with the elastic modulus generally ranging between 10 and 15 GPa. Degrees of conversion values are typically reported within the range of 55–75%, being influenced by curing protocols and material composition. These variations reflect the significant heterogeneity in experimental design, filler chemistry, and testing conditions, which limit direct cross-study comparisons but support consistent trends in the performance of bioactive systems.

A structured methodological approach is therefore essential for deriving clinically relevant conclusions regarding polymerization shrinkage dynamics and mechanical performance.

In addition, the limitations of the studies included should be considered when interpreting the reported findings. A large proportion of the available evidence is based on in vitro experimental designs, with variability in testing protocols, sample preparation, and evaluation methods, which may affect the comparability and generalizability of the results.

### 2.1. Study Design

This literature review examined bioactive filler technologies [bioactive glass (BAG), calcium phosphate (CaP), and surface-pre-reacted glass-ionomer (S-PRG)] incorporated into bulk-fill composite resins, focusing on polymerization shrinkage behavior and mechanical performance in posterior load-bearing restorations. This approach allowed for the synthesis of heterogeneous evidence from laboratory investigations, materials science analyses, and clinical studies addressing physicochemical and clinical implications of bioactive bulk-fill systems.

### 2.2. Literature Search Strategy

The search strategy combined controlled vocabulary terms and free-text keywords using Boolean operators (AND/OR), including combinations of “bulk-fill composite”, “bioactive fillers”, “bioactive glass”, “calcium phosphate”, “ACP”, “S-PRG fillers”, “polymerization shrinkage”, “shrinkage stress”, “degree of conversion”, “mechanical properties”, “wear resistance”, and “posterior restorations”. Database searches performed in PubMed/MEDLINE, Scopus, and Web of Science initially identified 412 records. After duplicate removal, 336 studies remained for title and abstract screening. Subsequently, 187 full-text articles were assessed for eligibility. Studies were selected according to their relevance to polymerization behavior, mechanical performance, ion-release mechanisms, bioactivity persistence, and clinical applicability of bioactive bulk-fill restorative systems. Additional references considered relevant for background context, mechanistic explanation, and foundational materials science concepts were identified through manual cross-referencing of bibliographies and citation tracking. Overall, 223 references were included in the final narrative synthesis.

The data selection process involved an initial screening of titles and abstracts to identify studies relevant to the scope of this review, followed by full-text evaluation. Articles were selected based on their relevance to polymerization behavior, mechanical performance, and bioactive properties of bulk-fill composites, with particular emphasis on studies providing quantitative or comparative data. Studies lacking sufficient methodological detail or not directly addressing the topic were excluded to ensure the scientific relevance and consistency of the analyzed data.

Because the currently available evidence is highly heterogeneous and predominantly based on laboratory investigations, a formal methodological quality or risk-of-bias assessment was not performed. Nevertheless, the limitations associated with heterogeneous experimental protocols, variability in filler formulations, non-standardized testing methodologies, and the scarcity of long-term clinical investigations were critically considered during data interpretation and synthesis.

Priority was given to studies published between 2020 and 2026 to capture recent developments in bioactive restorative materials, with seminal works from 2010 to 2017 included to establish foundational context. Reference lists of identified articles were manually reviewed to locate additional relevant publications.

### 2.3. Eligibility Criteria

Studies were selected if they examined bioactive bulk-fill composite systems and reported data on polymerization shrinkage, degree of conversion, mechanical properties (flexural strength, elastic modulus, and fracture resistance), or ion-release behavior. Both laboratory-based investigations and clinical studies relevant to posterior restorations were considered. Publications not addressing bulk-fill restorative systems or lacking quantitative data on shrinkage, conversion, or mechanical performance were excluded.

### 2.4. Data Extraction and Synthesis

Relevant findings from selected studies were synthesized qualitatively to identify relationships between bioactive filler composition, polymerization behavior, and mechanical performance. Extracted information included material composition, filler type and loading, shrinkage magnitude, degree of conversion, mechanical properties, ion-release kinetics, and bioactivity indicators (pH changes, ion leaching, and apatite formation potential). Evidence was organized into three thematic domains:

- Shrinkage and conversion kinetics as influenced by bioactive filler type;
- Mechanical property trade-offs (strength vs. stiffness);
- Bioactivity persistence and clinical relevance.

### 2.5. Quality Considerations and Limitations

The current evidence base is primarily derived from laboratory investigations employing heterogeneous experimental protocols, including variations in composite formulations, filler percentages, curing conditions, and testing methodologies. Long-term clinical studies evaluating bioactive bulk-fill systems remain limited (typically <12 months follow-up), restricting conclusions regarding shrinkage stress behavior and mechanical durability in posterior restorations. The interpretation of findings must therefore consider this experimental variability and the scarcity of long-term randomized clinical trials directly comparing bioactive bulk-fill restorative systems.

### 2.6. Polymerization Shrinkage in Bulk-Fill Composite Resins

Polymerization shrinkage remains one of the principal limitations affecting the clinical performance of resin-based composite restorations, particularly in posterior load-bearing applications [32,33]. Although bulk-fill composite resins were engineered to simplify restorative procedures through placement in thicker increments, quantifying shrinkage magnitude and stress development in bioactive bulk-fill systems remains a central objective in contemporary restorative material science [34].

#### 2.6.1. Mechanisms of Polymerization Shrinkage

During polymerization, the transition from weak van der Waals interactions to covalent bond formation is accompanied by progressive free volume reduction, as described by the Duda–Vrentas theory, leading to increased molecular packing and restricted segmental mobility [35,36]. As the degree of conversion increases, the mobility of reactive species becomes diffusion limited, significantly affecting polymerization kinetics. In this context, the interaction between long-chain radicals and more mobile short-chain radicals plays a critical role in termination mechanisms, as diffusion constraints reduce the probability of radical encounters and promote vitrification-controlled behavior [35,36].

**Figure 2:**
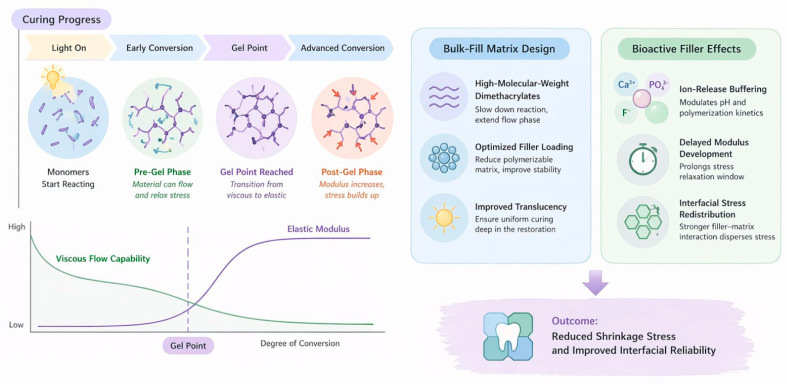
Schematic illustration of polymerization shrinkage stress development during curing of bulk-fill composite resins. The transition from pre-gel viscous flow compensation to post-gel elastic modulus increase governs stress generation, while bulk-fill matrix design and bioactive filler incorporation act through complementary mechanisms to reduce shrinkage stress.

Bulk-fill materials enable thicker placement (4–5 mm) through modified photoinitiator systems and altered network formation kinetics [37]. Stress-relieving monomer systems—including high-molecular-weight dimethacrylates and addition–fragmentation chain-transfer mechanisms—slow polymerization kinetics and reduce stress development without compromising degree of conversion [38]. This enables bulk-fill materials to maintain acceptable dimensional stability compared with conventional incremental systems [39]. Shrinkage stress development is closely related to viscoelastic behavior during polymerization’s pre-gel and post-gel phases [40], aspects illustrated in Figure 2.

In early curing stages, viscous flow partially compensates for volumetric contraction; however, once the material reaches the gel point and elastic modulus increases rapidly, further shrinkage generates internal stress transferred to the bonded tooth structure [41]. Polymerization kinetics and modulus development are therefore critical determinants of interfacial stress generation [42].

The incorporation of bioactive fillers—including bioactive glass (BAG), calcium phosphate (CaP), and surface pre-reacted glass-ionomer (S-PRG) particles—introduces distinct physicochemical interactions within the resin matrix. These interactions alter polymerization kinetics through filler–matrix interfacial effects, modify elastic modulus development during network formation, and consequently influence the shrinkage stress profile [43,44]. The specific mechanisms depend on filler chemistry, particle morphology, surface reactivity, and ion-release behavior under polymerization conditions [45,46].

#### 2.6.2. Shrinkage Stress Development in Deep Posterior Restorations

Polymerization shrinkage becomes particularly critical in deep posterior restorations, where the cavity geometry and bonded surface area directly influence stress accumulation at the tooth–restoration interface [47]. High configuration factor (C-factor) cavities (C-factor ≥ 5) restrict material flow during polymerization and concentrate stress within the composite and along adhesive interfaces, generating polymerization stress of 35–40 MPa in conventional systems [48], with bioactive formulations reducing this to 22–32 MPa [49].

When shrinkage stress exceeds adhesive bond strength, marginal gaps form and interfacial debonding occurs [50]. Shrinkage stress transmitted to surrounding tooth structures induces cuspal deflection, particularly in large Class I and Class II restorations [51]. Experimental studies on bulk-fill systems show that polymerization contraction contributes to enamel microcrack formation, postoperative sensitivity, and marginal leakage when stress compensation is insufficient [52,53]. This effect is especially relevant in posterior restorations under functional loading, where dimensional instability compromises long-term performance [54].

Beyond marginal degradation, shrinkage stress reduces internal adaptation and dentin bond integrity [55]. Variations in shrinkage stress behavior among bulk-fill formulations stem from differences in matrix composition, filler distribution, and polymerization rate [56]. These formulation-dependent factors are critical determinants of clinical outcomes [57].

#### 2.6.3. Strategies Used in Bulk-Fill Composites to Reduce Shrinkage Stress

The development of bulk-fill composites has focused on formulation strategies that reduce shrinkage stress while preserving mechanical performance [58]. Three principal approaches are employed:

(1) Organic Matrix Modification. High-molecular-weight dimethacrylates (urethane-based and bis-phenol A glycidyl dimethacrylate derivatives) combined with stress-relieving additives enable viscous flow compensation during the pre-gel phase, extending stress accommodation before gel-point transition [59,60].
(2) Increased Filler Loading and Optimization. Filler loading of 70–85% *w*/*w* with optimized particle size distribution reduces the relative volume of the polymerizable resin matrix. Since volumetric contraction occurs primarily in the organic phase, higher filler fractions lower shrinkage values (typically 1.0–2.0% vs. 2.5–3.0% conventional) and improve dimensional stability [61,62]. Pre-polymerized fillers and modified filler–matrix interfacial dynamics further contribute to stress redistribution during curing [63].
(3) Enhanced Translucency and Photoinitiator Efficiency. Improved material translucency and optimized photoinitiator systems enable homogeneous light transmission through deeper increments (4–5 mm), supporting adequate polymerization while limiting localized stress accumulation [64]. These combined material design strategies allow bulk-fill composites to achieve controlled shrinkage behavior despite increased increment thickness [65], supporting their clinical adoption in posterior restorative procedures [66].

#### 2.6.4. Bioactive Filler-Specific Mechanisms for Shrinkage Stress Mitigation

The incorporation of bioactive fillers into bulk-fill matrices introduces matrix-modifying effects distinct from conventional inert filler systems. Ion-releasing bioactive systems—particularly bioactive glass (BAG) and calcium phosphate (CaP) particles—generate localized pH changes (increasing pH from 5.5–6.0 to 7.0–7.5) and ion leaching (Ca^2+^, PO_4_^3−^, F^−^, and Sr^2+^) during and immediately after polymerization [67,68]. These ionic interactions may alter polymerization kinetics by modifying photoinitiator efficiency and free-radical availability, potentially extending the viscous flow phase and delaying stress development [69].

Additionally, bioactive fillers with reactive surface chemistries exhibit enhanced interfacial bonding with the resin matrix compared to conventional glass fillers [70]. Stronger filler–matrix interfaces may redistribute polymerization-induced stresses more efficiently throughout the composite volume, reducing localized stress concentrations at weak interfacial zones [71]. Surface pre-reacted glass-ionomer (S-PRG) particles, which carry a pre-formed glass-ionomer coating, demonstrate particularly robust filler–matrix interactions and may provide superior stress distribution characteristics [72].

However, the extent to which these mechanisms contribute to net shrinkage stress reduction rather than stress redistribution remains incompletely characterized. Quantitative comparative analyses of shrinkage stress behavior in bioactive versus conventional bulk-fill systems are limited, and interaction effects between bioactive filler ion release and bulk-fill monomer systems require further investigation [73,74].

Polymerization kinetics and degree of conversion play a critical role in shrinkage stress development, as higher conversion levels are generally associated with increased network rigidity and reduced capacity for stress relaxation [69]. The relationship between volumetric shrinkage, elastic modulus, and generated stress is inherently coupled with stress evolution depending not only on the magnitude of shrinkage but also on the rate of modulus development during curing [70]. As the material transitions from a viscous to a viscoelastic state, stress-relaxation mechanisms become increasingly important and can be described using classical viscoelastic models such as Maxwell and Kelvin–Voigt systems [40,57].

In this context, stress generation is influenced by the balance between polymerization rate and the ability of the material to dissipate stress through viscous flow and network rearrangement. Filler–matrix interactions play a key role, as the efficiency of stress transfer is dependent on interfacial bonding, typically governed by salinization [72]. Additionally, filler morphology, particle size distribution, and spatial arrangement affect polymerization dynamics by altering light transmission, radical mobility, and local conversion gradients [5,63].

Filler loading is another critical factor, as increased filler content generally reduces volumetric shrinkage but may accelerate modulus development, thereby increasing stress if relaxation mechanisms are insufficient. Bioactive fillers, such as bioactive glass, introduce additional complexity due to ion release and potential pH changes in the local environment, which may influence polymerization kinetics and network formation [43,44]. These effects differ significantly from those observed in ACP or S-PRG systems, highlighting the need for a mechanistic differentiation between filler types when evaluating their role in shrinkage stress mitigation [43,44].

The assessment of polymerization shrinkage stress is highly dependent on the experimental methodology employed. Common techniques include tensometry, strain gauge-based measurements, and digital image correlation (DIC), each providing different levels of spatial and temporal resolution. These methods are influenced by testing conditions, particularly the degree of constraint applied during polymerization, often expressed as a simulated C-factor, which significantly affects stress development. In addition, curing parameters such as light intensity, exposure time, and polymerization rate directly influence both shrinkage kinetics and stress evolution. The lack of standardized testing protocols across studies contributes to variability in reported values, highlighting the need for harmonized methodologies to enable more reliable comparisons and interpretation of shrinkage stress data.

The structure of the polymer network must also be considered when evaluating shrinkage stress behavior. Parameters such as cross-link density and network heterogeneity significantly influence mechanical performance and stress development. Highly cross-linked networks generally exhibit increased stiffness but reduced capacity for stress relaxation, while heterogeneous network formation may lead to localized stress concentrations. The incorporation of fillers further affects segmental mobility and may promote the formation of microdomains with distinct mechanical and physicochemical properties, influencing both polymerization kinetics and stress distribution.

Over time, additional factors contribute to the evolution of material properties. Hydrolytic degradation, ion leaching from bioactive fillers, and the formation of microvoids or porosity can alter the integrity of the polymer network. These processes may lead to gradual reductions in elastic modulus and mechanical strength, particularly under conditions of cyclic loading and prolonged exposure to the oral environment. Therefore, long-term stability must be considered when evaluating the overall effectiveness of bioactive systems for shrinkage stress mitigation.

Despite their beneficial effects, the incorporation of bioactive fillers may also introduce several limitations that must be considered. Ion-releasing systems, such as bioactive glass and ACP, can compromise the filler–matrix interface due to reduced silanization efficiency, potentially leading to decreased mechanical strength and increased susceptibility to hydrolytic degradation. In addition, enhanced water sorption associated with bioactive components may affect dimensional stability and accelerate material aging. The release of ions, while beneficial for remineralization, may also result in changes in mechanical properties over time, particularly under cyclic loading conditions. Furthermore, variations in filler size, distribution, and loading strategies can influence polymerization behavior and stress development in unpredictable ways, depending on the formulation. These trade-offs highlight the need for optimized material design to balance bioactivity with mechanical performance and long-term clinical reliability.

### 2.7. Mechanical Performance Requirements of Bulk-Fill Restorative Materials

Mechanical performance determines the clinical reliability of bulk-fill restorations in posterior load-bearing environments [75]. In addition to polymerization shrinkage behavior, properties such as flexural strength, elastic modulus, fracture resistance, and wear resistance play a central role in maintaining marginal adaptation, structural integrity, and functional stability of restorations placed in deep cavity configurations [76]. Evaluation of these mechanical parameters is essential when assessing the suitability of bulk-fill composite resins for stress-bearing posterior applications and the influence of emerging bioactive filler technologies on restorative performance [77].

#### 2.7.1. Flexural Strength of Bulk-Fill Composite Resins

Flexural strength represents one of the primary mechanical indicators of the ability of resin-based composite materials to resist deformation and fracture under functional loading conditions [78]. According to ISO 4049 requirements, restorative composite materials intended for occlusal load-bearing applications should demonstrate flexural strength values exceedingly approximately 80 MPa to ensure adequate clinical performance [79].

Contemporary bulk-fill composite resins generally exhibit flexural strength values comparable to those of conventional incremental composite systems, supporting their use in posterior restorations [80]. These improvements are attributed to advances in filler loading strategies, optimized particle size distribution, and modifications in resin matrix composition that enhance stress transfer between the organic matrix and reinforcing filler particles [81].

Consequently, the interaction between matrix composition, filler characteristics, and polymerization kinetics plays a critical role in determining overall mechanical reliability of bulk-fill restorative systems [82].

#### 2.7.2. Elastic Modulus and Stress Distribution Behavior

The elastic modulus of resin-based restorative materials represents a key parameter influencing stress distribution within restored teeth and surrounding structures [83]. Elastic modulus values approaching those of dentin improve stress distribution and reduce cuspal deflection [84].

Bulk-fill composite resins have been engineered to achieve balanced elastic modulus values that support both sufficient rigidity and controlled stress absorption during polymerization [85]. Modifications in monomer chemistry and filler architecture contribute to slower modulus development during curing, allowing for partial stress relaxation during the pre-gel phase and reducing shrinkage-induced stress transmission to cavity walls [86].

The elastic modulus is closely related to polymerization kinetics and volumetric shrinkage behavior, so optimization of stiffness development therefore represents an essential strategy for improving marginal adaptation and biomechanical compatibility in deep posterior restorations [87].

#### 2.7.3. Fracture Resistance in Posterior Restorative Applications

Fracture resistance represents a critical factor influencing long-term survival of posterior composite restorations subjected to cyclic occlusal loading [88]. The reinforcing capacity of bulk-fill composite resins depends not only on intrinsic material strength but also on their interaction with cavity configuration, adhesive interfaces, and polymerization shrinkage stress development [89].

Experimental investigations evaluating restored posterior teeth have demonstrated that bulk-fill composite systems may provide fracture resistance comparable to conventional incremental restorative techniques, particularly when shrinkage stress is effectively controlled through optimized matrix chemistry and filler architecture [90]. Reduced cuspal deflection and improved stress distribution within restored structures have been associated with enhanced structural stability in deep cavity restorations [91].

These findings highlight the importance of coordinated optimization between polymerization behavior and mechanical performance in restorative composite design [92].

#### 2.7.4. Wear Resistance Under Occlusal Loading

Wear resistance represents an essential mechanical property influencing the long-term performance of posterior composite restorations [93]. Occlusal surfaces are continuously exposed to mastication-related stresses and abrasive contact with antagonist teeth, so restorative materials must maintain surface integrity to preserve anatomical morphology and functional occlusion over time [94].

The wear behavior of bulk-fill composite resins is strongly influenced by filler composition, particle size distribution, filler–matrix interfacial bonding, and degree of conversion achieved during polymerization [95]. Improvements in filler technology and increased filler loading contribute to enhanced wear resistance in contemporary bulk-fill restorative systems, supporting their clinical application in stress-bearing posterior regions [96].

Importantly, the incorporation of functional and bioactive filler systems may further influence wear behavior through modifications in filler–matrix interaction and stress redistribution mechanisms. The contemporary literature documents wear resistance benchmarks in posterior composite restorations at approximately 20–50 μm per 100,000 masticatory cycles [97]. Ion-releasing fillers—including bioactive glass (BAG), calcium phosphate systems (CaP), and surface pre-reacted glass-ionomer (S-PRG) fillers—have demonstrated wear performance comparable to or exceeding conventional high-strength particulate fillers, with the literature reporting wear rates typically ranging between 22 and 50 μm per 100,000 cycles depending on filler composition and matrix architecture [98,99]. This evidence indicates that bioactive filler incorporation does not compromise surface durability. Optimized filler–matrix interactions may contribute to sustained mechanical performance and long-term restorative success in high-stress posterior applications [100].

### 2.8. Bioactive Filler Technologies in Bulk-Fill Composite Resins

Bioactive filler technologies represent an important development in contemporary bulk-fill composites by combining mechanical reinforcement with ion-release-mediated biological functionality at the tooth–restoration interface [101].

Unlike conventional reinforcing fillers that primarily improve strength and dimensional stability, bioactive particles actively interact with the surrounding oral environment through ion-exchange mechanisms supporting mineral deposition, buffering acidic conditions associated with cariogenic activity, and modulating free-radical polymerization kinetics through localized pH elevation and ionic interactions with the resin matrix [102]. These properties are especially relevant in bulk-fill restorative systems, where increased increment thickness and polymerization stress development require optimized filler–matrix interaction to maintain marginal adaptation and structural stability [103]. Among the most extensively investigated bioactive filler systems incorporated into contemporary restorative composite formulations are bioactive glass particles, calcium phosphate-based fillers, and surface pre-reacted glass-ionomer (S-PRG) fillers, each contributing differently to the structural and functional behaviors of bulk-fill restorative materials [104].

To facilitate a comparison between the principal bioactive filler systems currently investigated for bulk-fill restorative composite applications, their main compositional characteristics, ion-release profiles, and functional effects on restorative performance are summarized in Table 1.

#### 2.8.1. Key Distinctions Among Bioactive Filler Systems

Key distinctions among these three bioactive filler systems emerge when considering their mechanisms for modulating polymerization stress development [118]. Bioactive glass systems leverage ionic buffering to slow cross-linking kinetics during the pre-gel phase; calcium phosphate fillers achieve maximum stress reduction through matrix plasticization and delayed modulus development; and S-PRG fillers provide a balanced stress-modulation approach, combining ion-exchange buffering with structural reinforcement to achieve intermediate stress modulation while maintaining mechanical robustness. These mechanistic differences directly influence the suitability of each filler technology for specific restorative scenarios and clinical priorities [119].

#### 2.8.2. Bioactive Glass Fillers

Bioactive glass fillers represent one of the most extensively investigated classes of bioactive particles incorporated into resin-based restorative materials due to their ability to release calcium and phosphate ions and promote mineral deposition at the tooth–restoration interface [120]. These silica-based glass systems typically contain calcium, sodium, and phosphate components capable of participating in ion-exchange reactions when exposed to saliva or dentinal fluid, leading to the formation of hydroxycarbonate apatite-like layers that may contribute to remineralization processes and improved interfacial stability [121,122].

In addition to their remineralization potential, bioactive glass fillers modulate polymerization kinetics through ionic buffering. Released calcium and phosphate ions elevate local pH during early polymerization stages, slowing the progression of free-radical cross-linking reactions. This kinetic modulation permits partial stress relaxation in the pre-gel phase, reducing the maximum shrinkage-induced stress transmitted to cavity walls from approximately 35–40 MPa (conventional composites) to 22–28 MPa (BAG-enhanced systems) in high C-factor cavities [123]. Because these particles exhibit partial surface reactivity, their incorporation into composite formulations has been associated with buffering capacity under acidic conditions and improved resistance to marginal demineralization [124,125]. This buffering effect prolongs the pre-gel phase and facilitates stress relaxation before matrix rigidity is established [126]. Such effects are especially relevant in posterior restorations, where marginal degradation remains one of the main factors affecting restoration longevity [127,128].

However, incorporation of bioactive glass fillers must be carefully balanced with the mechanical requirements of bulk-fill restorative systems, as variations in particle size distribution, surface treatment, and filler loading may influence flexural strength and fracture resistance [129,130]. Contemporary formulation strategies therefore aim to optimize the proportion of bioactive glass particles in order to preserve structural reliability while maintaining therapeutic functionality [131,132].

#### 2.8.3. Calcium Phosphate-Based Fillers

Calcium phosphate-based fillers constitute a significant class of bioactive reinforcing fillers designed to facilitate mineral ion release and promote apatite crystallization within demineralized dental tissues [133]. This category encompasses amorphous calcium phosphate (ACP) particles, nanostructured calcium phosphate systems, and related derivatives that function as localized reservoirs of bioavailable calcium and phosphate ions, particularly under acidic microenvironmental conditions [134,135].

When incorporated into composite matrices, calcium phosphate fillers enhance remineralization capacity at restoration margins and throughout adjacent dentin, enabling restorative materials to actively participate in halting caries progression and stabilizing compromised tooth structure [136,137]. Beyond their mineralization potential, calcium phosphate fillers—particularly amorphous calcium phosphate (ACP)—undergo phase transformation during polymerization, inducing localized plasticization of the adjacent resin matrix. This effect delays elastic modulus development, permitting extended stress relaxation throughout the polymerization process and reducing maximum shrinkage-induced stress from approximately 35–40 MPa to 25–32 MPa in high C-factor cavities. CaP systems achieve the most pronounced reduction in polymerization stress among bioactive filler technologies, though at the cost of a slightly reduced post-cure elastic modulus compared to conventional glass-filled composites [138,139].

However, clinical implementation of calcium phosphate fillers in bulk-fill systems demands judicious compositional balance, as excessive filler loading compromises mechanical integrity due to modulus mismatch and suboptimal interfacial adhesion relative to conventional glass-based reinforcements [140,141]. Modern material engineering therefore prioritizes synergistic approaches that preserve ion-release bioactivity while maintaining the flexural strength and fracture resistance essential for reliable posterior performance [142,143].

#### 2.8.4. Surface Pre-Reacted Glass-Ionomer (S-PRG) Fillers

Surface pre-reacted glass-ionomer (S-PRG) fillers represent a sophisticated class of bioactive particles engineered to release multiple ions—including fluoride, strontium, sodium, borate, and silicate—thereby facilitating remineralization, pH buffering, and modulation of cariogenic bacterial behavior at the tooth–restoration interface [144]. These particles are synthesized through controlled surface reactions between fluoroaluminosilicate glass and polyacrylic acid, enabling stable integration into resin composite matrices while preserving sustained ion-release functionality [144,145].

The continuous ion release from S-PRG fillers strengthens interfacial stability and significantly reduces secondary caries risk through localized pH elevation and enhanced mineral deposition within adjacent dental tissues [146,147]. Notably, strontium ion release promotes remineralization of demineralized dentin and enamel, amplifying the therapeutic benefit of restorations incorporating this technology [148]. The ion-exchange mechanism of S-PRG fillers contributes to polymerization kinetics modulation, with released ions buffering pH during the curing phase and moderating cross-linking progression. This results in intermediate shrinkage stress reduction—from ~35–40 MPa to ~24–30 MPa in high C-factor cavities—while preserving the high elastic modulus (11–13 GPa) and flexural strength (100–135 MPa) characteristic of pre-reacted glass-ionomer fillers [149].

From a materials science perspective, S-PRG fillers uniquely combine structural reinforcement with bioactive functionality, rendering them particularly valuable for bulk-fill composite systems that must simultaneously deliver mechanical robustness and biological responsiveness [148,149]. This dual functionality highlights the growing role of filler chemistry in developing restorative materials capable of interacting with the oral microenvironment while maintaining structural integrity in posterior load-bearing applications [149,150].

### 2.9. Influence of Bioactive Fillers on Polymerization Shrinkage Behavior

Polymerization shrinkage remains a principal determinant of marginal adaptation and long-term clinical performance in resin-based composite restorations, particularly in bulk-fill systems placed within deep posterior cavities [151]. Although volumetric contraction is primarily governed by organic matrix composition and polymerization kinetics [151,152], accumulating evidence indicates that filler chemistry significantly influences stress development during the curing process [152,153].

Bioactive filler technologies have emerged as promising strategies for optimizing polymerization behavior through modifications in filler loading, matrix–filler interfacial interactions, and surface reactivity [154]. These physicochemical modifications are especially critical in bulk-fill restorative systems, where increased increment thickness amplifies shrinkage-induced stress concentrations and demands sophisticated stress-management strategies to preserve marginal integrity and structural reliability [154,155].

#### 2.9.1. Influence of Filler Loading on Volumetric Shrinkage Behavior

Volumetric shrinkage in resin-based composite materials occurs predominantly within the organic matrix during conversion of dimethacrylate monomers into a cross-linked polymer network [156]. Shrinkage stress behavior—the mechanical stress generated by this volumetric contraction—results from combined effects involving filler morphology, interfacial coupling efficiency, and polymerization kinetics. Consequently, increasing filler volume fraction represents one of the most effective strategies for attenuating shrinkage magnitude in contemporary restorative composite systems [156,157]. This principle is particularly relevant in bulk-fill materials, where increased increment thickness amplifies stress concentration at the tooth–restoration interface [158].

Bioactive fillers modulate shrinkage primarily through volumetric replacement of the polymerizable resin matrix [159]. The incorporation of reactive particles such as bioactive glass and calcium phosphate systems reduces the relative proportion of organic matrix while simultaneously modifying stress transfer mechanisms during polymerization [158,159]. The efficacy of shrinkage reduction depends critically on filler morphology, particle size distribution, and interfacial coupling efficiency [160]. Excessive filler loading without optimized silane surface treatment may paradoxically compromise reinforcement efficiency and stress-bearing capacity, despite apparent shrinkage-reduction benefits [161].

These findings indicate that filler loading represents a multifactorial parameter in shrinkage behavior. Volumetric replacement alone does not explain stress development in bioactive composite systems, and interfacial bonding plays an equally important role [162].

#### 2.9.2. Matrix–Filler Interaction Mechanisms and Polymerization Kinetics Modulation

**Figure 3:**
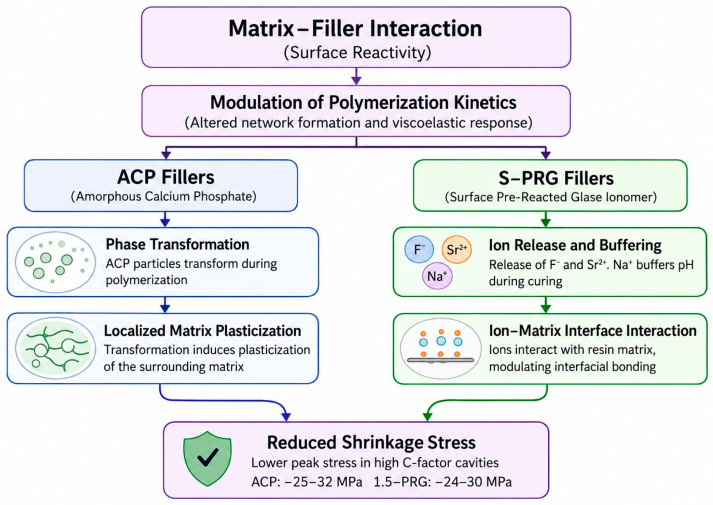
Matrix–filler interaction mechanisms influencing polymerization kinetics and shrinkage stress development in bulk-fill composite resins. Reactive ACP and S-PRG filler systems modulate network formation dynamics through phase transformation, ion release, and delayed elastic modulus development.

Beyond simple volumetric replacement of the organic matrix, bioactive fillers actively influence polymerization shrinkage behavior through modifications in polymer network formation kinetics [163]. The transition from pre-gel to post-gel polymerization phases represents a critical juncture in stress generation, and alterations in filler surface chemistry significantly modulate viscoelastic response during this transitional window [163,164]. These mechanisms are illustrated in Figure 3, which summarizes the influence of bioactive fillers on polymerization kinetics, stress development, and viscoelastic behavior during network formation.

The influence of bioactive fillers on polymerization shrinkage behavior and mechanical performance is governed by a complex interaction between filler chemistry, loading strategy, particle morphology, surface reactivity, and matrix–filler interfacial coupling. Filler chemistry directly affects ion-release kinetics, local pH modulation, and polymerization dynamics [163]. Bioactive glass systems release calcium and phosphate ions that can buffer acidic conditions and modify free-radical polymerization kinetics, whereas calcium phosphate fillers, particularly ACP systems, induce localized matrix plasticization and delayed elastic modulus development. In contrast, S-PRG fillers provide multivalent ion release combined with relatively stable mechanical reinforcement due to their pre-reacted glass-ionomer surface structure [164,165].

Filler loading protocols also play a critical role in shrinkage stress development. Increasing filler content reduces the relative proportion of the polymerizable resin matrix and therefore decreases volumetric shrinkage; however, excessive incorporation of reactive fillers may compromise stress dissipation by accelerating modulus development and reducing network flexibility. The balance between shrinkage reduction and mechanical reliability therefore depends not only on filler concentration but also on filler dispersion and interfacial bonding quality [166].

Reactive filler systems interacting with surrounding monomer matrices may reduce shrinkage stress not only through direct volumetric shrinkage reduction, but also through stress redistribution and viscoelastic stress-relaxation mechanisms during polymerization [165]. In particular, calcium-containing particles, especially ACP systems, may induce localized matrix plasticization and delayed elastic modulus development during the transition from pre-gel to post-gel phases. This prolonged viscoelastic response allows for partial stress dissipation before rigid polymer network formation occurs, contributing to lower measured shrinkage stress values in high C-factor bulk-fill restorations [165,166]. However, current evidence remains insufficient to determine whether the reported reduction in shrinkage stress primarily reflects true volumetric shrinkage mitigation or predominantly stress redistribution phenomena during curing.

Particle morphology and surface characteristics further influence stress evolution during polymerization. Smaller particles and optimized particle size distributions improve stress distribution and enhance filler packing efficiency, while irregular or poorly dispersed particles may generate localized stress concentrations and conversion heterogeneity [164,165]. Surface reactivity and silanization efficiency are particularly important because the filler–matrix interface governs stress transfer between the polymer network and reinforcing particles. Inadequate silane coupling may reduce interfacial adhesion, increase water sorption, and accelerate hydrolytic degradation under oral conditions [70,71,92]. These interactions become especially relevant in high C-factor bulk-fill restorations, where restricted material flow limits stress compensation during polymerization. Under these conditions, stress accumulation is strongly influenced by the combined effects of polymerization kinetics, modulus development, interfacial bonding efficiency, and viscoelastic stress-relaxation capacity. Consequently, bioactive filler systems should not be evaluated solely according to ion-release behavior or shrinkage reduction but rather through an integrated analysis of matrix–filler interactions, stress redistribution mechanisms, and long-term mechanical stability under functional loading conditions.

Similarly, S-PRG fillers modulate interfacial bonding characteristics through ion-mediated interactions at the filler–matrix interface. Released ions—particularly fluoride (F^−^), strontium (Sr^2+^), and sodium (Na^+^)—buffer pH during the curing phase and facilitate ion-exchange reactions with the resin matrix, extending the pre-gel phase and moderating cross-linking progression. This ion-mediated kinetics modulation results in intermediate shrinkage stress reduction—from approximately 35–40 MPa to 24–30 MPa in high C-factor cavities—while preserving elastic modulus (11–13 GPa) and flexural strength (100–135 MPa) characteristics essential for posterior load-bearing applications [167]. These observations identify interfacial chemistry as a key determinant of shrinkage stress development in bulk-fill restorative systems [167,168].

The principal mechanisms by which bioactive fillers influence polymerization shrinkage behavior are synthesized in Table 2.

#### 2.9.3. Contribution of Ion Release and Buffering Capacity to Interfacial Stress Stabilization

Beyond their influence on polymerization kinetics, bioactive fillers indirectly enhance shrinkage-related performance through ion-release-mediated stabilization of restoration margins [169]. Bioactive glass and S-PRG fillers release calcium, phosphate, fluoride, and strontium ions that promote mineral deposition and buffer acidic microenvironments at the tooth–restoration interface [170,171].

Although these mechanisms do not directly reduce volumetric contraction during curing, they significantly improve marginal resistance to degradation processes associated with shrinkage-induced gap formation and microleakage [172,173]. This buffering activity proves particularly critical in deep posterior restorations, where stress concentration and marginal breakdown represent principal risk factors for secondary caries development [174].

Ion-release-mediated interfacial stabilization represents a complementary pathway through which bioactive fillers mitigate long-term consequences of polymerization shrinkage stress, extending restoration longevity beyond what mechanical stress reduction alone can achieve [175].

#### 2.9.4. Experimental Evidence on Shrinkage Stress Behavior in Bioactive Bulk-Fill Systems

Recent experimental investigations evaluating shrinkage stress in restorative composites incorporating ion-releasing fillers demonstrate shrinkage stress values ranging from 22–32 MPa, substantially lower than conventional bulk-fill systems (35–40 MPa), contingent upon filler type, matrix composition, and filler architecture [176]. These benefits derive from synergistic contributions of elevated filler loading (typically 60–80 wt.%), modified polymerization kinetics through ion release and pH buffering, and enhanced stress redistribution during the critical pre-gel to post-gel transition phase [177,178].

However, the reported shrinkage stress values should not be interpreted as universally reproducible parameters, as substantial variability exists among the currently available studies. Differences in experimental methodologies, including tensometry, strain gauge analysis, and digital image correlation, may significantly influence the magnitude and temporal profile of the recorded stress values. In addition, specimen geometry, cavity configuration factor (C-factor), bonded surface area, curing protocols, light intensity, exposure duration, and aging conditions directly affect polymerization kinetics and stress development during network formation.

Variability in filler loading, particle morphology, silanization efficiency, and matrix composition further complicates direct comparison between bioactive restorative systems. Moreover, most currently available evidence is derived from laboratory-based investigations performed under simplified and non-standardized conditions that do not fully reproduce the dynamic oral environment. Consequently, methodological heterogeneity and differences in testing protocols may introduce bias when comparing shrinkage stress behavior and mechanical performance across studies, and the currently reported numerical ranges should therefore be interpreted with caution.

The shrinkage-modulating potential of bioactive fillers proves formulation dependent rather than universally applicable across all material systems [179]. Variations in silane coupling efficiency, filler morphology, particle size distribution, and resin matrix composition substantially influence stress development behavior, underscoring the necessity for material-specific evaluation when assessing shrinkage performance of bioactive composite technologies [180]. However, standardized inter-material comparisons remain limited.

Representative experimental observations from the peer-reviewed literature documenting shrinkage-related performance of ion-releasing filler systems are consolidated in Table 3.

#### 2.9.5. Translational Implications for Shrinkage Stress Control in Bulk-Fill Restorative Systems

From a translational perspective, the incorporation of bioactive filler technologies into bulk-fill composite resins represents a promising strategy for improving marginal adaptation and long-term interfacial stability in posterior restorations affected by polymerization shrinkage stress [187]. Although volumetric contraction remains primarily governed by resin matrix composition, interaction between reactive filler particles and polymer network formation kinetics contributes to improved stress redistribution during curing [188,189].

Filler systems capable of releasing calcium, phosphate, fluoride, and strontium ions stabilize restoration margins through buffering activity and promotion of mineral deposition within adjacent dental tissues [190,191]. These mechanisms prove particularly relevant in deep cavity configurations, where polymerization stress accumulation compromises adhesive interface integrity [192]. The incorporation of bioactive filler technologies into bulk-fill restorative systems enhances biological responsiveness and dimensional stability during early restoration maturation stages [193].

### 2.10. Influence of Bioactive Fillers on Mechanical Performance of Bulk-Fill Composite Resins

The incorporation of bioactive fillers into bulk-fill composite resins introduces new opportunities for enhancing restorative material performance beyond traditional reinforcement strategies based exclusively on inert filler systems [194]. Because posterior restorations encounter complex mechanical loading conditions during mastication, rigorous evaluation of how ion-releasing filler technologies influence structural reliability remains essential for determining clinical applicability [195,196].

The contribution of individual factors to the overall mechanical behavior is interdependent and varies depending on material formulation and curing conditions. Filler loading is generally considered a dominant factor, as it directly reduces volumetric shrinkage; however, increased filler content may also accelerate modulus development, potentially leading to higher stress if relaxation mechanisms are insufficient [109,160]. In contrast, the polymer network structure, including cross-link density and degree of conversion, primarily governs the balance between stiffness and the capacity for stress dissipation [156,163]. Materials with higher conversion typically exhibit increased rigidity but reduced ability to relax internal stresses [8,9].

Filler–matrix interactions represent another critical component, as the efficiency of stress transfer depends on interfacial bonding quality and silanization [70,71]. Poor interfacial coupling may reduce mechanical performance but can also locally affect stress distribution. Additionally, filler morphology and spatial distribution influence light transmission, polymerization kinetics, and local conversion gradients, further contributing to mechanical variability [72,92].

Although these factors are widely recognized as key determinants of mechanical performance, their relative quantitative contribution is not consistently reported across studies due to differences in experimental design and testing conditions. As a result, the available evidence supports a mechanistic hierarchy of influence rather than precise numerical weighting, with filler loading and network structure typically exerting the most significant effects.

Beyond their biological functionality, bioactive fillers influence flexural strength, elastic modulus, fracture resistance, and wear behavior through modifications in filler architecture, matrix–filler interaction mechanisms, and polymer network formation dynamics [197]. Consequently, systematic evaluation of mechanical implications associated with bioactive filler incorporation represents a critical step in determining the clinical performance and long-term reliability of bioactive bulk-fill restorative systems [198].

#### 2.10.1. Influence of Bioactive Fillers on Flexural Strength and Structural Reliability

Flexural strength represents one of the primary indicators of structural reliability in resin-based restorative composite materials intended for posterior load-bearing applications [199]. Because this property reflects resistance to deformation and crack initiation under functional loading conditions, it critically determines whether incorporation of bioactive fillers can be achieved without compromising restorative durability [200].

Moderate incorporation of bioactive glass fillers generally preserves flexural strength when reactive particles are combined with conventional reinforcing filler systems [201]. Similar behavior has been reported for calcium phosphate-based fillers, where optimized particle distribution and controlled filler loading maintain mechanical performance comparable to conventional bulk-fill formulations [202,203]. These findings indicate that hybrid filler architectures combining reinforcing and bioactive particles represent an effective strategy for maintaining structural reliability while introducing therapeutic functionality [204]. However, excessive incorporation of ion-releasing fillers may reduce reinforcement efficiency because reactive particles typically exhibit lower stiffness compared with traditional silanized glass fillers [205]. Contemporary bulk-fill material design emphasizes balanced filler composition to maintain flexural strength values compatible with posterior stress-bearing applications [206,207].

#### 2.10.2. Influence on Elastic Modulus and Fracture Resistance Behavior

Elastic modulus represents a critical parameter influencing stress distribution within restored teeth, particularly in deep posterior restorations where polymerization shrinkage stress and occlusal loading act simultaneously [208]. Composite materials exhibiting elastic modulus values approaching those of dentin promote homogeneous stress transfer across the tooth–restoration interface and reduce cuspal deflection risk [208,209].

Bioactive filler incorporation influences stiffness development through modifications in filler composition and matrix–filler interaction mechanisms [210]. Calcium-containing filler systems, including calcium phosphate and S-PRG technologies, facilitate controlled modulus development during curing, supporting improved stress redistribution within restored tooth structures [211]. Because fracture resistance depends on both intrinsic material strength and stress transfer efficiency across adhesive interfaces, these modifications enhance biomechanical compatibility in bulk-fill systems placed in deep cavity restorations [211,212].

Experimental studies evaluating restored posterior teeth demonstrate that bulk-fill composites incorporating optimized hybrid filler architectures maintain fracture resistance comparable to conventional incremental restorative techniques when filler–matrix interaction is appropriately controlled [213]. These observations underscore the importance of coordinated optimization between polymerization behavior and mechanical performance when developing therapeutic restorative composite materials [214]. The principal mechanical effects associated with bioactive filler incorporation in bulk-fill composite resins are consolidated in Table 4.

#### 2.10.3. Influence of Bioactive Fillers on Wear Resistance and Long-Term Surface Stability

Wear resistance represents an essential determinant of long-term performance in posterior composite restorations exposed to repeated occlusal contact and abrasive interaction with antagonist teeth [221]. Preservation of surface morphology is critical for maintaining functional occlusion and reducing marginal degradation over time [222,223].

The wear behavior of bulk-fill composite resins incorporating bioactive fillers depends primarily on filler particle size distribution, interfacial bonding characteristics, and overall filler architecture within the composite matrix [224]. Although the incorporation of reactive ion-releasing particles may influence surface hardness depending on formulation characteristics, optimized hybrid filler systems maintain wear resistance comparable to conventional bulk-fill restorative materials [225,226].

These findings demonstrate that appropriate integration of bioactive fillers within reinforcing filler networks preserves surface durability while introducing therapeutic functionality capable of supporting long-term restorative stability in posterior load-bearing environments [227]. Wear resistance does not represent a limiting factor for clinical adoption of bioactive bulk-fill restorative systems when material design is appropriately optimized [228]. Overall, S-PRG systems provide the most balanced mechanical–bioactive profile, whereas CaP systems provide the greatest shrinkage stress reduction but show formulation-dependent stiffness variability.

## 3. Discussion

The incorporation of bioactive filler technologies into bulk-fill composite resins represents a relevant advancement in restorative material design, as these systems influence polymerization shrinkage behavior through mechanisms that extend beyond conventional matrix volume reduction. Reactive filler systems actively modulate polymerization kinetics and stress redistribution during network formation in addition to their reinforcing function. This observation indicates that filler chemistry represents a primary variable controlling dimensional stability in restorative composite formulations.

A major challenge in bioactive bulk-fill development lies in reconciling biological functionality with mechanical performance. Ion-releasing fillers offer significant advantages in terms of remineralization capability and pH buffering at the tooth–restoration interface; however, their incorporation may compromise stiffness development and mechanical reliability depending on their composition and interfacial interactions. Hybrid filler architectures represent an effective strategy for addressing this limitation, allowing for the combination of reinforcing particles with bioactive components while maintaining structural integrity and introducing biological responsiveness.

Posterior restorations in deep cavity configurations represent a particularly relevant application for bioactive bulk-fill materials, as stress accumulation at adhesive interfaces constitutes a primary failure mechanism. Ion-releasing fillers capable of supporting mineral deposition and stabilizing pH conditions may mitigate shrinkage-induced gap formation and marginal degradation; however, long-term clinical validation of these effects remains limited.

Despite encouraging laboratory findings, evidence supporting the long-term clinical reliability and translational applicability of bioactive bulk-fill composite systems remains limited. Most currently available investigations are based on in vitro experimental models or short-term clinical evaluations, with relatively few studies extending beyond 12–24 months of follow-up. Although several clinical investigations have reported acceptable short- to medium-term performance of bulk-fill restorative systems in posterior applications, definitive evidence demonstrating superior long-term behavior of bioactive formulations compared with conventional composites remains insufficient. In addition, substantial variability persists regarding shrinkage-stress evaluation methodologies, including differences in cavity configuration, constraint conditions, curing protocols, aging procedures, and fatigue-loading simulation. Similarly, long-term in vivo evidence regarding sustained ion-release durability, bioactivity persistence, and maintenance of remineralization potential under functional oral conditions remains scarce. Consequently, current translational claims should be interpreted cautiously until more standardized long-term clinical investigations and fatigue-aging studies become available.

In addition to initial polymerization behavior and short-term mechanical performance, the long-term stability of bioactive bulk-fill composite systems is strongly influenced by interfacial and environmental factors that remain insufficiently standardized across the available literature [61,62,91]. The efficiency of silanization and the quality of filler–matrix interfacial adhesion play a critical role in stress transfer, crack propagation resistance, and preservation of mechanical integrity over time. In bioactive systems, particularly those containing reactive ion-releasing fillers such as bioactive glass and ACP particles, incomplete or unstable silane coupling may reduce interfacial bonding efficiency and increase susceptibility to hydrolytic degradation under oral conditions [205,209].

Time-dependent viscoelastic phenomena, including creep and stress relaxation, must also be considered when evaluating the clinical behavior of these restorative systems. Although delayed modulus development and increased stress relaxation may contribute to lower polymerization shrinkage stress during curing, excessive viscoelastic deformation may negatively affect long-term dimensional stability and resistance to occlusal loading [157,184]. These effects become particularly relevant in posterior restorations exposed to repetitive masticatory forces and high cyclic loading conditions.

Furthermore, water uptake and ion release may progressively alter the polymer network structure and filler–matrix interface. Increased water sorption can promote plasticization of the resin matrix, reduce cross-link density, cause filler debonding, and cause microcrack formation, thereby accelerating mechanical degradation and surface wear over time [146,147]. Chemical degradation processes associated with hydrolysis, pH fluctuations, enzymatic activity, and continuous ion exchange may further contribute to reductions in the elastic modulus, flexural strength, and interfacial stability during long-term clinical service [147,153,158].

Importantly, substantial variability exists among experimental studies regarding evaluation conditions. Reported mechanical and shrinkage-related outcomes are highly dependent on testing parameters, including storage medium, aging duration, temperature, humidity, thermal cycling protocols, and applied loading conditions [70,148]. In many studies, mechanical testing is performed under simplified laboratory conditions that do not fully reproduce the complex oral environment characterized by fluctuating temperature, moisture exposure, pH variations, and dynamic masticatory loading [152]. Consequently, direct comparison between studies remains difficult, and the long-term clinical implications of bioactive filler incorporation should be interpreted with caution until more standardized aging and fatigue-testing methodologies become available.

Although flexural strength, elastic modulus, and wear resistance are frequently reported as independent parameters, these properties are strongly interconnected and should be interpreted collectively when evaluating the mechanical behavior of bioactive bulk-fill composite systems [77,197]. Reductions in polymerization shrinkage stress are often associated with modifications in filler loading, polymer network structure, and filler–matrix interactions, which simultaneously influence stiffness development, fracture behavior, and long-term surface durability.

In general, increasing bioactive filler content contributes to shrinkage reduction by decreasing the relative proportion of polymerizable resin matrix; however, excessive incorporation of reactive fillers may compromise reinforcement efficiency and reduce the elastic modulus due to lower intrinsic stiffness and suboptimal interfacial coupling compared with conventional silanized glass fillers [32,33,34]. Consequently, materials exhibiting superior stress-relaxation capacity do not necessarily demonstrate optimal mechanical rigidity or wear resistance.

Calcium phosphate-based systems, particularly ACP-containing formulations, appear to provide the greatest reduction in polymerization stress through delayed modulus development and matrix plasticization mechanisms [127,128]. Nevertheless, this stress-relaxation behavior may also contribute to reduced stiffness and increased susceptibility to surface degradation under cyclic occlusal loading. In contrast, S-PRG-based systems generally demonstrate a more balanced mechanical profile, preserving elastic modulus and wear resistance while still providing moderate shrinkage stress reduction through ion-mediated kinetic modulation and improved stress redistribution [135,137,166]. Bioactive glass systems occupy an intermediate position, where shrinkage reduction and remineralization potential are achieved simultaneously, although mechanical performance remains highly dependent on filler loading strategy and interfacial bonding efficiency [118].

Wear resistance is also affected by the combined interaction of filler morphology, degree of conversion, cross-link density, and interfacial stability [97]. Materials with insufficient filler–matrix coupling or increased water sorption may exhibit accelerated surface degradation despite favorable initial shrinkage behavior [222,223].

Importantly, the relationship between shrinkage stress reduction and preservation of mechanical performance should not be interpreted as universally synergistic across all bioactive filler systems. Although several formulations demonstrate reduced shrinkage stress values together with clinically acceptable mechanical behavior, important trade-offs may occur depending on filler chemistry, loading strategy, and interfacial coupling efficiency. In particular, calcium phosphate-based systems, especially ACP-containing formulations, may reduce the elastic modulus and increase formulation-dependent mechanical variability due to delayed modulus development and matrix plasticization mechanisms occurring during polymerization.

Furthermore, lower measured shrinkage stress values do not necessarily indicate direct reduction of volumetric shrinkage itself. In many bioactive systems, the observed stress reduction may instead reflect redistribution of polymerization-induced stresses, delayed elastic modulus development, prolonged viscoelastic relaxation, or improved stress dissipation during the transition from pre-gel to post-gel phases. Consequently, the distinction between true shrinkage mitigation and stress redistribution remains an important unresolved aspect in the interpretation of polymerization behavior in bioactive bulk-fill restorative systems.

Current trends in restorative material science support the development of multifunctional systems integrating controlled polymerization behavior with sustained ion release and enhanced interfacial compatibility. Innovative approaches including rechargeable bioactive fillers capable of re-releasing ions after topical fluoride application and adaptive polymer matrices with pH-responsive cross-linking kinetics represent promising directions for developing restorative materials that engage dynamically with the biological environment. Despite promising laboratory evidence, long-term clinical validation of bioactive bulk-fill systems remains limited. Future research should prioritize standardized shrinkage stress testing protocols, long-term ion-release stability evaluation, and optimization of hybrid filler architectures balancing mechanical reinforcement and sustained bioactivity.

## 4. Conclusions

Bioactive filler technologies significantly influence the polymerization behavior and mechanical performance of bulk-fill composite resins through matrix–filler interactions, ion release, and modulation of polymerization kinetics. Current evidence indicates that bioactive systems reduce polymerization shrinkage stress from approximately 35–40 MPa in conventional composites to 22–32 MPa in optimized formulations, corresponding to stress reductions of nearly 25–40%.

Among the investigated systems, calcium phosphate fillers provide the greatest stress-relaxation potential, whereas S-PRG fillers demonstrate the most balanced mechanical–bioactive profile, maintaining flexural strength values generally above 100 MPa and elastic modulus values within approximately 11–13 GPa. Bioactive glass systems additionally contribute to remineralization and interfacial stabilization through ion-release-mediated buffering effects.

Although wear resistance of optimized bioactive bulk-fill systems remains within clinically acceptable ranges, long-term stability may still be influenced by hydrolytic degradation, water sorption, filler–matrix interfacial degradation, and cyclic loading conditions. Despite promising laboratory evidence, current findings remain limited by heterogeneous experimental methodologies and insufficient long-term clinical data.

Future research should prioritize standardized shrinkage-stress evaluation protocols, long-term aging studies, and optimization of filler–matrix interfacial chemistry to improve the balance between bioactivity, shrinkage control, and mechanical durability in posterior restorative applications.

## Figures and Tables

**Table 1:** Overview of bioactive filler systems in bulk-fill resin composites.

Filler Type	Typical Composition	Ion-Release Profile	Biological Effects	Influence on Restorative Performance	Limitations
Bioactive glass	Silica-based glass containing Ca, Na, and P [105]	Ca^2+^, PO_4_^3−^	Remineralization support [106]; buffering capacity [107]	Improved marginal stability [108]; resistance to demineralization [107,108]	May influence mechanical strength depending on filler loading [109]
Calcium phosphate fillers	ACP, nano-CaP derivatives [110]	Ca^2+^, PO_4_^3−^	Apatite formation [110]; mineral reservoir effect [110,111]	Support remineralization at restoration margins [112]	Reduced stiffness compared with conventional reinforcing fillers [113]
S-PRG fillers	Fluoroaluminosilicate glass (surface reacted) [114]	F^−^, Sr^2+^, Na^+^, BO_3_^3−^, SiO_4_^4−^	Antibacterial activity [114]; buffering effect and remineralization [115]	Enhanced interfacial stability; secondary caries prevention potential [116]	Composition-dependent mechanical influence [117]

**Table 2:** Mechanistic pathways governing shrinkage stress modulation in bioactive bulk-fill systems.

Filler Type	Primary Mechanism	Stress Reduction (Conv. 35–40 MPa → Bioactive)	% Reduction	Reported Clinical Effects
Bioactive Glass	Matrix volume replacement; ionic exchange at interface; pH buffering in pre-gel phase [153]	22–28 MPa [154]	~38%	Enhanced marginal stability; decelerated cross-linking kinetics [154]
Calcium Phosphate Fillers	ACP phase transformation; matrix plasticization; delayed elastic modulus development [155,156]	25–32 MPa [157]	~29%	Maximum stress relaxation; extended stress compensation throughout curing [157]
S-PRG Fillers	Multivalent ion release (F^−^, Sr^2+^, Na^+^); ion-mediated kinetic modulation; preserved mechanical properties [158,159]	24–30 MPa [160]	~24–29%	Balanced stress redistribution; conserved elastic modulus (11–13 GPa); flexural strength (100–135 MPa) [160]

**Table 3:** Representative experimental observations regarding the influence of bioactive fillers on polymerization shrinkage behavior in restorative composite systems.

Study Focus	Filler System	Shrinkage Stress (Conv. vs. Bioactive)	% Reduction	Mechanism
Bioactive Glass Incorporation	Silica-based glass with Ca^2+^/PO_4_^3−^ release	38 MPa → 24 MPa	~37% [181]	Matrix dilution + ionic exchange at interface + pH buffering decelerating cross-linking kinetics [182]
ACP-Based Fillers	Amorphous calcium phosphate (nano-scale)	40 MPa → 28 MPa	~30% [183]	ACP phase transformation + matrix plasticization + delayed elastic modulus development + extended stress relaxation in pre-gel phase [184]
S-PRG Fillers	Fluoroaluminosilicate glass (F^−^, Sr^2+^ release)	37 MPa → 27 MPa	~27% [185]	Ion-mediated kinetic modulation + buffering capacity + ionic exchange interactions + conserved elastic modulus (11–13 GPa) + flexural strength (100–135 MPa) [186]

**Table 4:** Influence of bioactive filler systems on mechanical performance parameters of bulk-fill composite resins.

Filler System	Flexural Strength	Elastic Modulus	Fracture Resistance	Clinical Implication
Bioactive glass fillers	Maintained when combined with reinforcing fillers [210]	Slight stiffness variation depending on formulation [211,212]	Comparable to conventional systems in hybrid matrices [213]	Structural reliability preserved with added remineralization potential
Calcium phosphate fillers	Formulation-dependent behavior [215]	Moderate modulus adjustment possible [216]	Maintained with optimized filler distribution [217]	Supports mineral stabilization at restoration margins [218]
S-PRG fillers	Comparable to conventional bulk-fill composites [219]	Improved stress redistribution behavior [218,219]	Favorable structural stability reported [220]	Enhanced interfacial durability and buffering capacity [220]

## Data Availability

All information analyzed in this review was obtained from previously published studies available in the scientific literature. No original datasets were generated.

## Abbreviations

The following abbreviations are used in this manuscript:

ACP	Amorphous Calcium Phosphate
AFCT	Addition–Fragmentation Chain Transfer
BAG	Bioactive Glass
CaP	Calcium Phosphate
C-factor	Configuration Factor
DIC	Digital Image Correlation
ISO	International Organization for Standardization
MPa	Megapascal
GPa	Gigapascal
S-PRG	Surface Pre-Reacted Glass Ionomer
